# Invariant Image-Based Currency Denomination Recognition Using Local Entropy and Range Filters

**DOI:** 10.3390/e21111085

**Published:** 2019-11-06

**Authors:** Hafeez Anwar, Farman Ullah, Asif Iqbal, Anees Ul Hasnain, Ata Ur Rehman, Peter Bell, Daehan Kwak

**Affiliations:** 1 Interdisciplinary Center for Digital Humanities and Social Sciences, Friedrich-Alexander-Universität Erlangen-Nürnberg, 91052 Erlangen, Germany; hafeez.anwar@fau.de; 2 Department of Electrical & Computer Engineering, COMSATS University Islamabad-Attock Campus, Attock 43600, Pakistan; farmankttk@cuiatk.edu.pk (F.U.); dr.ataurrehman@cuiatk.edu.pk (A.U.R.); 3 Department of Information and Communication Engineering, Inha University, Incheon 22212, Korea; asifsoul@inha.ac.kr; 4 EPAS Engineering, Topi 23460, Pakistan; anees@epas.com.pk; 5 Department of Computer Science, Kean University, Union, NJ 07083, USA

**Keywords:** image entropy, image processing, image segmentation

## Abstract

We perform image-based denomination recognition of the Pakistani currency notes. There are a total of seven different denominations in the current series of Pakistani notes. Apart from color and texture, these notes differ from one another mainly due to their aspect ratios. Our aim is to exploit this single feature to attain an image-based recognition that is invariant to the most common image variations found in currency notes images. Among others, the most notable image variations are caused by the difference in positions and in-plane orientations of the currency notes in images. While most of the proposed methods for currency denomination recognition only focus on attaining higher recognition rates, our aim is more complex, i.e., attaining a high recognition rate in the presence of image variations. Since, the aspect ratio of a currency note is invariant to such differences, an image-based recognition of currency notes based on aspect ratio is more likely to be translation- and rotation-invariant. Therefore, we adapt a two step procedure that first extracts a currency note from the homogeneous image background via local entropy and range filters. Then, the aspect ratio of the extracted currency note is calculated to determine its denomination. To validate our proposed method, we gathered a new dataset with the largest and most diverse collection of Pakistani currency notes, where each image contains either a single or multiple notes at arbitrary positions and orientations. We attain an overall average recognition rate of 99% which is very encouraging for our method, which relies on a single feature and is suited for real-time applications. Consequently, the method may be extended to other international and historical currencies, which makes it suitable for business and digital humanities applications.

## 1. Introduction

In this paper, we propose an image-based framework for the denomination recognition of Pakistani currency notes of the 2005 series. Such systems are important as they are used at several situations such as automatic vending machines and supporting visually impaired people [1] to identify the denomination of a given currency note. Consequently, such image-based denomination recognition of currency notes has become an active area of research where the proposed solutions can be divided into two main groups. The first group of methods [2] deals with currency images that are acquired with scanners. Such methods are more suitable for applications like automatic teller machines (ATM). However, the most common variations that are found in scanned images are the ones induced by the variations in currency notes orientation, scale and position. The second group of methods deals with currency note images that are taken with a camera in a cluttered environment. Such a system installed on a smart phone can be helpful for visually impaired people in their daily life. However, these methods face challenges due to the image variations caused by non-uniform illumination, background clutter, and partial occlusions.

The proposed solutions are broadly divided into three different groups, where the first group uses the so-called local features matching such as scale-invariant feature transform (SIFT) [3]. The second group makes use of supervised machine learning algorithms such as artificial neural networks (ANNs) [4]. Lastly, the third group of methods uses pure image processing techniques such as template matching [5].

**Figure 1:**
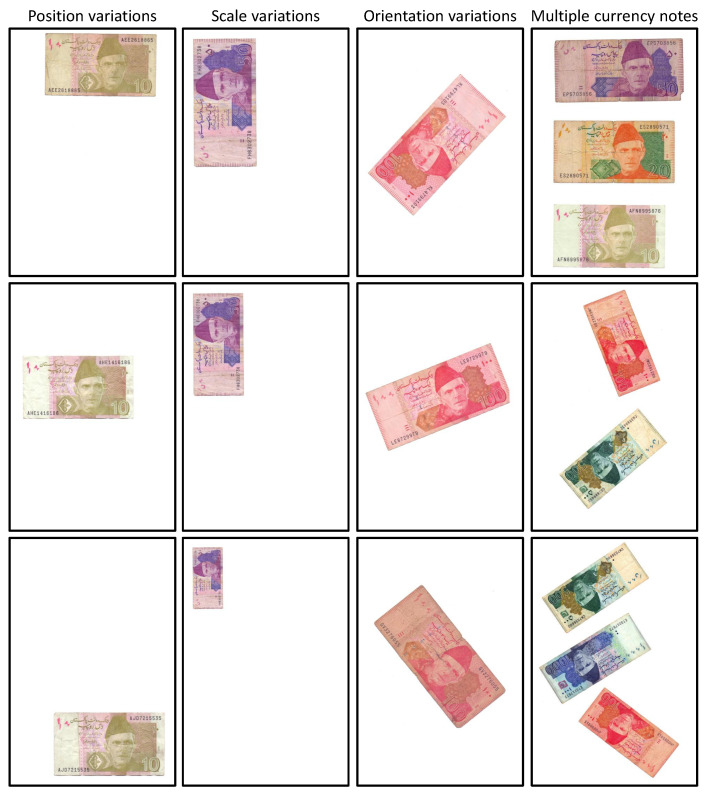
Common image variations found in currency note images.

**Figure 2:**
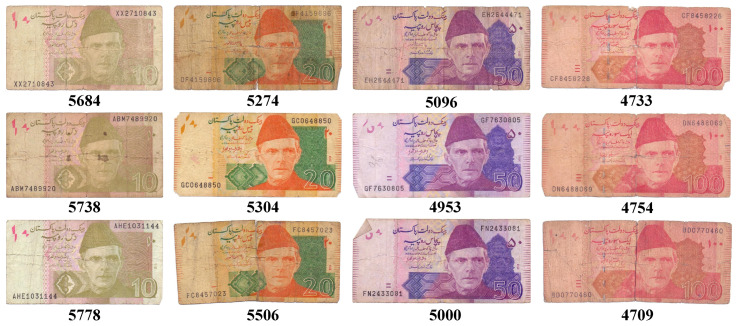
Variations in aspect ratios due to irregular currency note boundaries. The aspect ratio of each currency note is shown below it.

We take a different approach to the problem of image-based denomination recognition of scanned currency notes. We are interested in utilizing simple image filtering, rather than using a complex handcrafted local image descriptor or a complicated machine learning algorithm such as a convolutional neural network (CNN). In addition to simplicity, such filtering is also invariant to the most commonly found variations in the currency notes images. These image variations are not explicitly identified and dealt with in any of the previously proposed methods. Figure 1 depicts the variations that are found in the currency note images. The orientation differences found among the currency notes of any given denomination can cause variations in their images. This makes images of the same currency note look different from one another. This can clearly be observed in the third column of Figure 1. Similarly, the variations caused by position and scale differences of the currency notes induce variations in their images which are depicted in the first and second columns of Figure 1. Lastly, the image variations can also be caused by multiple currency notes in a single image as shown in fourth column of Figure 1. Apart from image variations, the recognition rate is also likely to be affected by the variations caused by the condition of currency notes themselves. The standard aspect ratios of currency notes are based on the freshly printed and unused notes as shown in Table 1. However, the excessive and rough use of the currency notes causes wear and tear, due to which, their boundaries become irregular. Since, we propose to use the aspect ratio as a single feature for recognizing these notes, such irregularities in the boundaries induce variations in the aspect ratios as well. This is demonstrated in Figure 2 where currency notes of multiple frequently used denominations are shown. It can be observed that the aspect ratios of currency notes belonging to a single denomination vary from one another. However, it should be noted that the measurements are based on our proposed image-based method. To summarize, in order to achieve an accurate denomination recognition of currency notes, it is important to address these variations. This has already been proved in other object categories such as ancient coins [6], and butterflies and fish [7], where image variations caused by changes in object orientation, scale, and position are very common.

### Related Work

Based on their approaches, the proposed solutions for currency denomination recognition can broadly be divided in the following three classes.

Machine learning-based methods:

The first class of methods uses machine learning techniques such as artificial neural networks (ANN) and support vector machines (SVMs). For instance Takeda and Omatu [4] used a mask of slabs that is convoluted with a local pixel neighborhood such that the coefficients of the masks are randomly chosen to be either 1 or 0. The aggregated results of several such convolutions are then provided as input to a three layered neural network (NN). Similarly, in Frosini et al. [8], the light refracted from the bank notes was captured with arrays of opto-electronic sensors and then provided to a multi-layered perceptron. Takeda and Nishikagi [9] extracted the information of the currency notes via the so-called axis symmetric masks and then provided it to an ANN. However, more recently, a state-of-the-art performing convolutional neural network (CNN) was used by Pham et al. [10] where they reported a result of 100% on an image dataset of 64,000 images belonging to 64 classes. In addition to the neural networks and its variants, other machine learning algorithms such SVM were also used along with its various types of kernels. In Chang et al. [11], the features constructed from sensors were used to represent the key regions of a currency note to predict whether it is real or fake. The task of classification is performed by a support vector machine (SVM) where different kernels were evaluated for classification accuracy. Similarly, He et al. [12] used principle component analysis (PCA) features to represent an edge image of a single banknote. A genetic algorithm (GA) was further used for feature selection. Such image representation was then used to train an SVM model, and later for testing it. Other machine learning algorithms used for currency recognition include the hidden Markov model (HMM) [13], k nearest neighbors (kNN) [14] and Gaussian mixture model (GMM) [15].

Local feature matching-based methods:

A local feature descriptor is a compact representation of the pixel intensity distribution within a local image patch. As a common practice, the feature descriptor is an N dimensional vector. For object recognition via feature matching, the local features such as scale-invariant feature transform (SIFT) [3] are extracted from the representative images of a given object and stored in a database. Then, from a given image, local features are extracted and matched with the ones using a distance measure such as the Euclidean distance. The local features of the stored image that are the nearest to the local features of the test image are declared to belong to the same object class. The local feature matching is also used for currency recognition. SIFT and its color variant also known as color SIFT are used for currency recognition by [16]. Similarly, Hasanuzzaman et al. [1] used speedup robust features (SURF) for currency recognition. More recently, Yousry et al. [17] used binary local feature descriptor oriented FAST and rotated BRIEF (ORB) to represent the input image and then compared with the representative images in the database using the Hamming distance. Other local features used for currency recognition include local binary patterns (LBP) [18] and a gray-level co-occurrence matrix (GLCM) [19].

Image processing-based methods:

The image processing based methods are purely based on pixel level manipulation techniques such as extracting the region of interest (ROI) of a banknote and then applying various measures such as the correlation [20]. Such methods are more relevant for real-time applications where relatively less processing resources are available. Similarly, Youn et al. [5] used the banknote size information along with multiple-template matching for a multiple currency recognition system.

The proposed paper currency recognition method is based on a two step process. The first step deals with the extraction of paper currency note from the image via local entropy and range filters. The extracted note region is then normalized and rotated to achieve scale-, translation-, and rotation-invariance. The second step is recognition of the extracted and processed region. This is done by computing the aspect ratio of the extracted region as currency notes differ from one another based on this feature. Since, the aspect ratio of a rectangular object is not affected by the scale, position, and in-plane orientation of the object, it is also invariant to these transformations. Hence, our proposed paper currency recognition method is invariant to changes in scale, position, and in-plane orientations.

## 2. Methodology

The proposed method is inspired by an automatic ancient coin segmentation [21]. Following their method, we also segment a currency note that is imaged on a homogeneous background by the following assumptions.

1. The image region depicting a currency note contains the highest information content.
2. The most rectangular object found in the image is a currency note that has a predefined but slightly varying aspect ratio depending on its condition.

Therefore, the segmentation becomes a two step process. The first step deals with the extraction of image regions with the highest information contents. The second step consists of finding rectangular regions among the extracted ones and calculating their aspect ratios. In the following, we elaborate on both of these steps.

### 2.1. Informative Region Extraction

Since, the currency note is imaged on a homogeneous background, the image region depicting the note is likely to have more variations in terms of pixel values than the background. We employ a local image neighborhood processing strategy to extract informative regions. The local image neighborhood is simply a subset of image pixels arranged in rows and columns. The image filters that process a local image neighborhood are called the local filters. In our proposed method, we use the following two local filters.

Local entropy filter:

Entropy gives the measurement of information content in an event, signal, or in our case, an image. Concretely, entropy is inversely proportional to the probability of a random variable where it is maximum if the value of probability is close to zero and vice versa. In the context of image, the information content is represented by the pixel intensity values that range from 0 to L−1 where L=256. The histogram of pixel intensity values [22] represents the number of pixels per intensity value as shown in Equation (1).

(1) $$h\left(i\right)=n_{i}\;\;\;\;i\in 0,1,2,\ldots ,255.$$

Each bin in this histogram gives the count of that particular intensity value as $n_{0},n_{1},\ldots ,n_{\left(L-1\right)}$. In other words, the total number of pixels in the local neighborhood having intensity value *i*, is $n_{i}$.

In order to convert this count into their respective probabilities, the histogram of intensity values is normalized by dividing the value of each bin over the total number of pixels in the local neighborhood. For instance, if the total number of rows in a local neighborhood is *M* and the total number of columns is *N*, then, the normalized histogram is given as,

(2) $$p\left(i\right)=h\left(i\right)/\left(M*N\right)=n_{i}/\left(M*N\right),\;\;\;\;i\in 0,1,2,\ldots ,255$$

where, p(0),p(1),…,p(255) give the probabilities of each intensity value in the local neighborhood.

The entropy of a local neighborhood Ω in an image is then found via this normalized histogram using Equation (3),

(3) $$H\left(\Omega \right)=-\sum_{i=0}^{255}p\left(i\right).\log_{2}\left(p\left(i\right)\right).$$

Local range filter:

The range of a local neighborhood is simply calculated by finding the difference of maximum intensity value in that particular neighborhood and the minimum.

**Figure 3:**
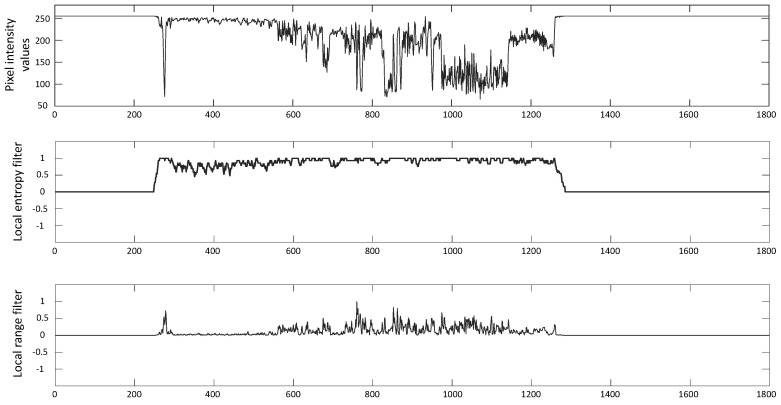
Responses of local entropy and range filters on a single row of currency note images (where the values at *x*-axis show the number of rows).

To summarize, the response of local entropy filter for homogeneous image regions will be minimum whereas it will be maximum for those regions having higher variations of pixel intensity values. A similar result will be achieved for the local range filter. Figure 3 shows the same effect where we depict the responses of both the filters on a one dimensional signal that is a single row of a currency note image. The flat part of the intensity signal corresponds to the homogeneous background while its fluctuating part shows the variations in pixel intensity values of the currency note. Consequently, both the local filters achieve results that are adequate for both the homogeneous and fluctuating part. It can be observed that they do not respond to the homogeneous part while their responses are more pronounced to the part of signal representing the pixel intensity variations.

**Figure 4:**
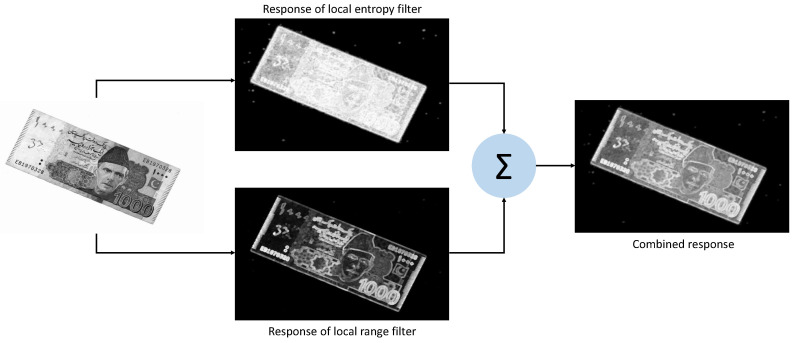
Responses of local entropy and range filters on a currency note image.

Sum of both filters:

Both these local filters are applied to a given note image. For each filter, the size of a local circular neighborhood is empirically selected as 3. The resultant response of each filter is scaled to the range between 0 and 1. Finally, both the responses are summed to get the combined response of both the filters. The individual and cumulative responses of both filters are shown in Figure 4. The region of images where both the filters give high response shows more informativeness than the background. However, the border region of the currency note in the combined response is more pronounced, which proves supportive at the stage of segmentation.

### 2.2. Currency Note Segmentation and Recognition via Aspect Ratio

**Figure 5:**
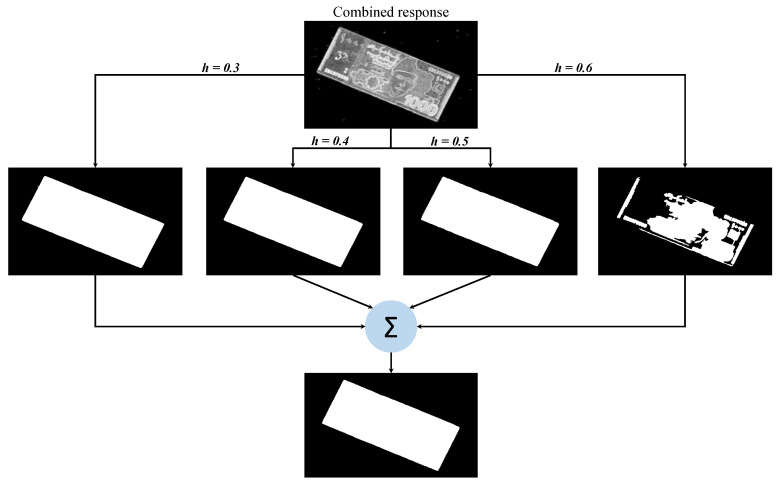
The process of segmentation mask generation using threshold-defined binarization masks.

For the current imaging conditions, a set of empirically defined thresholds (0.3, 0.4, 0.5, and 0.6) is applied to the combined response image for segmenting the currency note region. All the binarized images produced by each threshold are summed to get the final segmentation mask for the currency note. This whole procedure is shown in Figure 5. The resulting binarized masks generated by applying each threshold are very similar except for the last one. This effect is more or less observed on all the images of the dataset. As a last step, the aspect ratio of the generated mask is calculated to determine the denomination of currency note. To this end, we evaluated two kinds of aspect ratios for each denomination that are further explained in Section 4.

## 3. A New Pakistani Currency Notes Dataset (PCND)

We collect an image dataset of Pakistani notes with images having high variations with respect to in-plane orientation and position. These images are taken with a desktop flatbed scanner where the background is completely homogeneous. All the seven denominations of the Pakistani Currency of the 2005 series are represented in the dataset. We divide our dataset into two disjoint subsets. The first subset that we call the “training set” is used to calibrate the aspect ratio for each note. This is further elaborated in Section 4. We call the second dataset the “test dataset”, where currency notes of various denominations are scanned at arbitrary positions and orientations in the following three different settings.

1. This is the simplest setting with only one currency note per image.
2. The images in this setting contain two currency notes of different denominations. However, the notes are separated from each other to such an extent that they are not overlapped. Overlapping will cause their boundaries to merge thus resulting in a wrong segmentation. For this setting, the notes of consecutive denominations are chosen as they are more likely to get confused.
3. Finally, three notes per image of consecutive denominations are imaged together.

It should be noted that the images with multiple notes are simply made by combining the single note images such that they are scaled, rotated and positioned arbitrarily. Table 2 shows the total number of images per denomination in both subsets of the image dataset.

## 4. Results and Discussion

Since the proposed currency note recognition method is based on the aspect ratios of the banknotes, as a first step, the values of aspect ratios for each denomination have to be established. We simply divide the shorter side (width) of the note over its longer side (length) and then scale the number by multiplying it with 10,000. For instance the standard width of the currency note of 10 rupees is 65 mm and its standard length is 115 mm. Therefore, its aspect ratio is 10,000×(65/115), which is 5652. We adapt the following two different methods to obtain the aspect ratio for each denomination.

1. Standard aspect ratio: This is the aspect ratio of an original and new currency note.
2. Calibrated aspect ratio: The majority of the notes used in the market undergo wear and tear due to their age and usage. These currency notes are not in their original shape and thus their aspect ratios are more likely to differ from the new ones. We use currency note images in the training set to “calibrate” the aspect ratio for each denomination. To this end, we find the aspect ratios of currency notes for each denomination in the training set that include both new and used notes. The mean of all these aspect ratios is then considered as the aspect ratio of the respective denomination.

Both the standard and the calibrated aspect ratios for each denomination are shown in Table 3. The difference between standard and calibrated aspect ratios for each denomination can be observed. This is due to the fact that training set consists of used currency notes whose aspect ratios vary from one another due to the deterioration in their shapes. Once, the aspect ratios are established for each note type, the next step is to use them to label a given test note image. From a given image, the note image is extracted and then its aspect ratio is calculated using the proposed method. This aspect ratio is then compared with the aspect ratios of all the notes and the one nearest to it is assigned the label.

**Figure 6:**
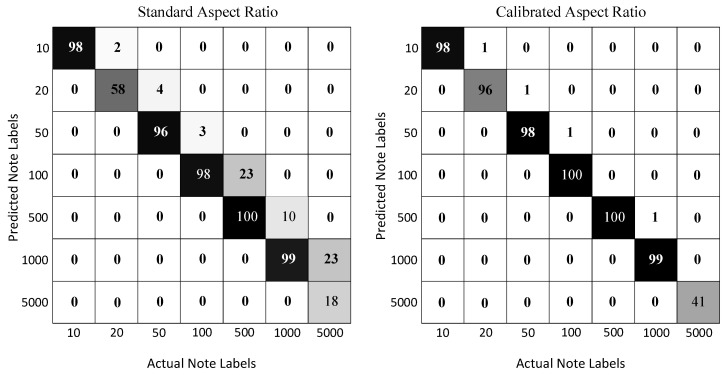
Confusion matrices for both the standard and calibrated aspect ratios.

**Figure 7:**
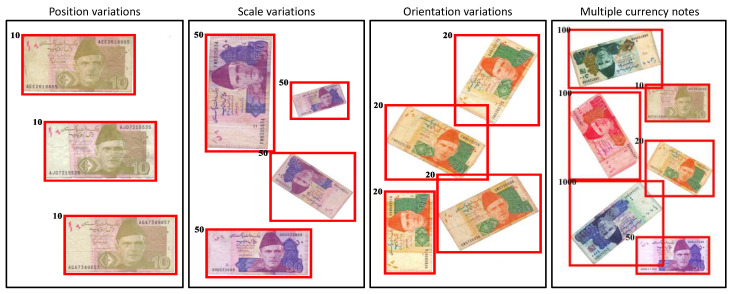
Visualization of denomination recognition of currency notes imaged at various positions, scales and orientations.

The images per note type vary from 60 to 100, for a total of 598 images. In each test image, there is a single instance of a note that is displayed at an arbitrary scale, position, and orientation. The results for both the methods are shown in Table 4 while the confusion matrices for each note type are shown in Figure 6. The aspect ratios based on calibration clearly outperform the standard aspect ratios. This is quite realistic as the notes in the test image data contain both new and old notes. Due to this reason, the calibration-based aspect ratios give flexible values for both new and old notes to be recognized. The denomination recognition results for currency notes images with different variation are shown in Figure 7. Our method successfully recognizes denominations of each currency note under the translation, scale, and rotation variations. It also very accurately recognizes the denomination of multiple currency notes that are imaged together. However, a failure on the currency note of 500 can be observed where it is wrongly recognized as a note with the denomination of 100.

## 5. Conclusions and Future Work

We performed denomination recognition of Pakistani currency notes from their images. Such recognition was achieved despite the image variations that are commonly found in currency images. These image variations are mainly caused by changes in currency note position, scale, and orientation. To achieve a denomination recognition that is invariant to these image variations, a two step process was proposed where, in the first step, informative regions from the images are extracted via local entropy and range filters. As a second step, these regions are extracted via binarization and their aspect ratios are calculated for final denomination recognition. The aspect ratio for each denomination is established by using a training set where, for each denomination, notes are used with various degrees of deterioration based on their usage. The proposed method is evaluated on a novel dataset of Pakistani currency notes of the 2005 series, where it achieves a recognition rate of 99% on a total of 598 images. The usage of a single feature, i.e., aspect ratio, for recognition makes our method more feasible for real-time application which we plan to test in the future by implementing this method on a Raspberry Pi.

## Figures and Tables

**Table 1:**
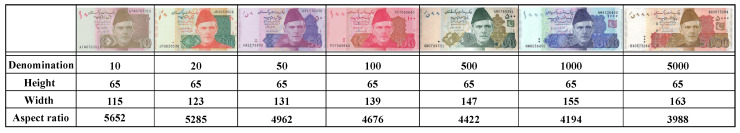
The Pakistani currency notes of the 2005 Series along with their width, height, and aspect ratios. The aspect ratio is calculated as (height/width).×10,000.

**Table 2:** Details of image dataset.

Number of Images per Denomination			
Training Set		Test Set	
Denomination	No. of Images	Denomination	No. of Images
10	105	10	100
20	101	20	97
50	101	50	96
100	94	100	97
500	101	500	77
1000	101	1000	90
5000	9	5000	41

**Table 3:** Values of standard and calibrated aspect ratios for different denominations.

	10	20	50	100	500	1000	5000
Standard aspect ratio	5652	5285	4962	4676	4422	4194	3988
Calibrated aspect ratio	5750	5356	5055	4762	4526	4295	4083
Difference	98	71	93	86	104	101	95

**Table 4:** Denomination recognition rates achieved on standard and calibrated aspect ratios where the recognition rates of calibrated aspect ratios (in bold) are better than those of the standard aspect ratios.

	10	20	50	100	500	1000	5000	Overall
**Standard aspect ratio**	100%	97%	96%	97%	77%	90%	41%	89%
**Calibrated aspect ratio**	100%	**99%**	**98%**	**99%**	**100%**	**99%**	**100%**	**99%**
